# Neural Strategies for Selective Attention Distinguish Fast-Action Video Game Players

**DOI:** 10.1007/s10548-012-0232-3

**Published:** 2012-05-22

**Authors:** Lavanya Krishnan, Albert Kang, George Sperling, Ramesh Srinivasan

**Affiliations:** Department of Cognitive Sciences, University of California, Irvine, CA 92617 USA

**Keywords:** Spatial attention, EEG, SSVEP, Video games, Brain networks

## Abstract

We investigated the psychophysical and neurophysiological differences between fast-action video game players (specifically first person shooter players, FPS) and non-action players (role-playing game players, RPG) in a visual search task. We measured both successful detections (hit rates) and steady-state visually evoked EEG potentials (SSVEPs). Search difficulty was varied along two dimensions: number of adjacent attended and ignored regions (1, 2 and 4), and presentation rate of novel search arrays (3, 8.6 and 20 Hz). Hit rates decreased with increasing presentation rates and number of regions, with the FPS players performing on average better than the RPG players. The largest differences in hit rate, between groups, occurred when four regions were simultaneously attended. We computed signal-to-noise ratio (SNR) of SSVEPs and used partial least squares regression to model hit rates, SNRs and their relationship at 3 Hz and 8.6 Hz. The following are the most significant results: RPG players’ parietal responses to the *attended* 8.6 Hz flicker were predictive of hit rate and were positively correlated with it, indicating attentional signal enhancement. FPS players’ parietal responses to the *ignored* 3 Hz flicker were predictive of hit rate and were positively correlated with it, indicating distractor suppression. Consistent with these parietal responses, RPG players’ frontal responses to the *attended* 8.6 Hz flicker, increased as task difficulty increased with number of regions; FPS players’ frontal responses to the *ignored* 3 Hz flicker increased with number of regions. Thus the FPS players appear to employ an active suppression mechanism to deploy selective attention simultaneously to multiple interleaved regions, while RPG primarily use signal enhancement. These results suggest that fast-action gaming can affect neural strategies and the corresponding networks underlying attention, presumably by training mechanisms of distractor suppression.

## Introduction

Selecting a subset of information from a sensory scene is possibly mediated by multiple neural mechanisms and strategies. These neural mechanisms, collectively termed ‘attention’, allow the selection of information defined either by location and/or feature. During visual search constrained to specific locations, attention can mediate this selection of information, both by enhancing the information at relevant locations (Carrasco et al. 2004; Hillyard et al. 1998) and by suppressing the information at irrelevant locations (Serences et al. 2004). These two attentional mechanisms, ‘stimulus enhancement’ and ‘distractor suppression’ can work in conjunction or alone, resulting in a net sensory gain of the relevant information, thereby bringing about its selection.

Multiple studies have shown the advantage of distractor suppression as a neural strategy when the sensory scene is noisy or when it contains multiple, interleaved distractors (Dosher and Lu 2000; Awh et al. 2003; Gobell et al. 2004; Serences et al. 2004). Awh et al. (2003) showed that spatial cueing effects were greater when the subjects expected a noisy display as opposed to when they expected a noiseless display. This effect held only if the subsequent target display was actually noisy and disappeared when it was noiseless. The result implied that when a subject expected a noisy stimulus, the strategy was to suppress the unattended locations, which would then lead to greater decrease in performance if the cue turned out to be invalid. Using a similar behavioral paradigm in an fMRI study, Serences et al. (2004) demonstrated that the preparatory activity in the posterior visual cortex was greater when the distractors surrounding the target locations were probable compared to when they were improbable. The fact that this observed effect was larger when the subsequent target image contained distractors, suggested the role of the preparatory activity in suppressing the information at the unattended locations.

Using a novel search task that required the deployment of attention to one, two, three or six regions, Gobell et al. (2004) investigated the spatial distribution of visual attention over multiple non-contiguous regions. Subjects were asked to identify the position of a single target that appeared in one of the attended locations, while ignoring ten false targets distributed across the unattended regions. Thus, in order for performance to be maintained in such a task, subjects were required to suppress the information at the unattended locations. The results showed that subjects could deploy attention to multiple non-contiguous locations in space by suppressing the interleaved distracting information. However, performance decreased as the number of regions increased, suggesting a decrease in attention modulation and therefore suppression with increasing number of locations. The authors used Fourier Theory to describe number of locations as spatial frequency of the stimuli and modeled attentional modulation as a low pass spatial filter, with higher gain at lower spatial frequencies.

These results suggest that suppression is available as a strategy for visual selection, but it is not apparent that all observers automatically deploy suppression. For one thing, in low noise displays with few distractors, subjects make use of only enhancement of the attended features/locations. Moreover, the studies described above were all conducted on extensively trained subjects with prior knowledge of the distractors, who may only have been able to employ distractor suppression after the training (Gobell et al. 2004). Dosher and Lu (1998) demonstrated that perceptual learning in an orientation discrimination task led to lowered thresholds mediated both by the reduction of internal additive noise (or stimulus enhancement) and by external noise exclusion (or distractor suppression). A study on working memory conducted by Berry et al. (2009), demonstrated that with practice, subjects were better able to filter out irrelevant information. This improvement in performance was accompanied by the suppression of event related potential (ERP) P1 amplitude, corresponding to the distractors. The result was interpreted as a demonstration of a decrease in the sensory gain associated with the distracting information (Luck and Hillyard 1995).

A question that follows from these results is whether the general extent of visuo-spatial training determines whether the subject makes use of suppression as a neural mechanism for selective attention. Behavioral studies have shown reliable advantage of professional sports training on perceptual abilities such as covert spatial attention, selective attention and spatial orienting ability (Nougier et al. 1992; Kioumourtzoglou et al. 1998). Recent psychophysical studies conducted by Green and Bavelier (2003, 2006, 2007) have demonstrated that fast-action gamers have an enhanced ability to selectively process and respond to visual information compared to non-gamers. This enhanced ability can be attributed to the fact that fast action games inherently require the quick processing of, and responses to, multiple objects in the visual field. In order to be able to quickly and correctly process a subset of information, attention has to be deployed to the relevant locations and/or features. The authors showed that fast action gamers have an increased attentional capacity, by using an enumeration task. The fast action gamers performed better than non-gamers, being able to subitize larger numbers (Green and Bavelier 2003). They also showed that fast action gaming enhances the spatial distribution of visual attention throughout the visual field by demonstrating the enhanced target localization ability of fast action gamers, both at the center and periphery (Green and Bavelier 2007). The most interesting result from these studies was the establishment of a causal relationship between fast-action gaming experience and enhanced visuo-spatial attention. This was achieved by training a group of non video game players on a fast action game for 10 days, and observing significant increases in performance not only in the video game but in other visual tasks as well. The neural basis of such enhanced perceptual abilities of fast action gamers is, however, relatively unknown. It is moreover unclear if the advantage shown by fast action gamers results from an increased ability to enhance relevant information or if they are better at suppressing irrelevant but distracting information. At the time of writing this, we are aware of only one other study (Mishra et al. 2011), which investigates the neural basis of the enhanced performance of fast action gamers by comparing the neural responses of action video game players and those who did not play any video games. Mishra et al. (2011), provide evidence for distractor suppression as being partly responsible for the enhanced performance of fast action gamers. The authors found that the response to the unattended flickering stimuli was reduced to a greater extent in video game players, relative to responses to the attended stimuli. However, the mechanism of the neural control of this distractor suppression was not investigated.

To investigate the relationship between suppression and the extent of visuo-spatial training, we used two groups of subjects, one group which extensively played fast action games, specifically first person shooter games (FPS players) and another which extensively played role-playing games (RPG players). The RPG players served as our control group since they had the similar video-game playing experience as the FPS players, without enjoying the potential training benefits in attentional filtering of FPS games. We used a modified version of the search paradigm employed by Gobell et al. (2004) to probe the differences between the two groups of gamers. We chose this paradigm because, by design, the task requires the subject to suppress irrelevant information presented at locations interleaved with task relevant locations. Our task required the subject to simultaneously attend to 1, 2 or 4 regions on an annulus, the number of regions to be attended and ignored produces quantifiably variable demands on attention modulation.

To simultaneously measure whole-brain responses to both the attended and unattended information, we designed an EEG experiment incorporating the *frequency tagging* design. Frequency tagging is an experimental design that has been used to study large-scale networks that could be mediating the selection and possible suppression of information in visual attention tasks (Ding et al. 2006; Morgan et al. 1996; Muller et al. 1998). Steady state visually evoked neural responses (SSVEP) are measured specific to each of the flickering stimuli presented simultaneously in the visual field, by presenting each stimulus at a different temporal frequency. In this way, we can monitor separate cortical responses to unattended and to attended regions of the display.

We examined the performance differences between FPS and RPG players, as the number of attended and unattended regions was varied. The low pass spatial filter characteristic of attention (Gobell et al. 2004) indicates that performance will decrease as the number of attended and unattended regions increases. We expect the FPS players to demonstrate higher performance than the RPG players as the attentional load (number of attended/unattended regions) increases (Green and Bavelier 2003) to reflect the enhanced spatial resolution of FPS players’ attentional filtering.

We obtained corresponding neurophysiological measures of steady state visually evoked potentials (SSVEPs) and computed the signal to noise ratio (SNR) at each stimulus frequency. From our previous studies (Ding et al. 2006; Srinivasan et al. 2006) we anticipated finding functionally distinct networks to be entrained at different tag frequencies. We modeled the SNR and hit rate data at 3 and 8.6 Hz using partial least squares regression (PLS). Within this model, we used the SNR responses to the attended or unattended flickering search arrays to predict the average hit rate in a trial. The direction of correlation between the SNR and hit rate was used to determine the nature of the neural mechanisms mediating performance. A *positive* correlation of SNR responses at the temporal frequency of the *attended* search array with hit rate indicates signal enhancement. This is because a larger network response to the attended locations would suggest increased processing of the attended locations (to enhance stimulus information). A *positive* correlation of SNR at the temporal frequency of the *unattended* search array with hit rate indicates distractor suppression suggesting increased processing of the unattended locations (to suppress stimulus information). We found that the two groups of gamers differed in their use of stimulus enhancement and distractor suppression, suggesting that visuo-spatial training influences the neural strategies employed to successfully select and respond to a subset of visual information in space.

## Methods

### Participants

Twenty-four subjects participated in this study, twelve played FPS games and twelve played RPG. To be included in either group, it was required that the participants be 18 years or older, play at least 4 days a week, 1 h each day and have normal or corrected vision. The FPS players (12 participants, 11 male, 1 left handed) played for an average of 9.08 h a week and had been playing for an average of 10.41 years, while the RPG players (12 participants, 9 male, 2 left handed) played for an average of 15.83 h, for an average of 11.67 years. The RPG players served as a control for this study, since they spent as much–if not more–time playing video games, but the RPG players would not be expected to enjoy the attention benefits that were specifically due to playing FPS games.

### Stimuli

**Fig. 1:**
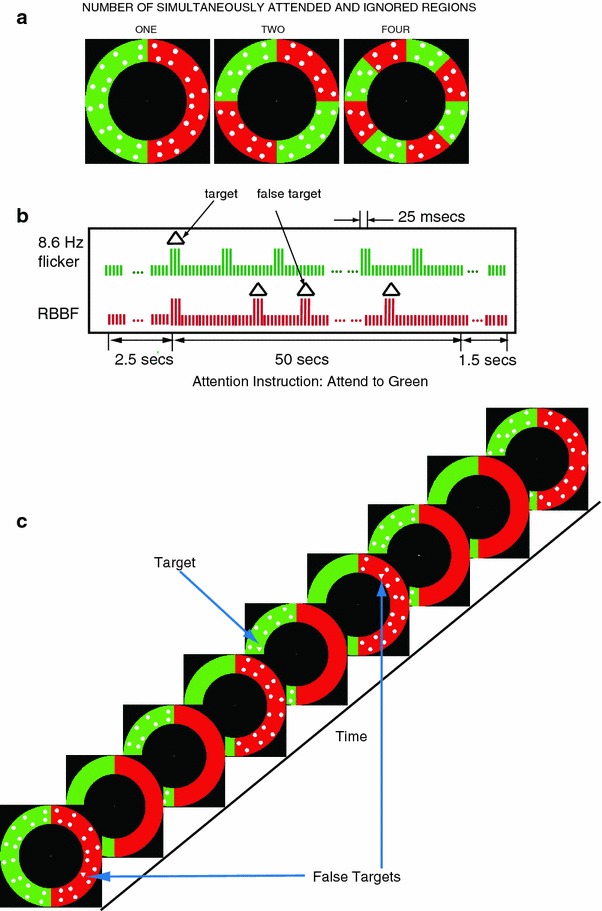
Illustration of the stimuli and of flicker generation. **a** The three spatial stimulus configurations. Only distractors (*dots*) are shown, no targets or false targets. **b** Flicker generation (8.6 Hz, *green*), random broadband flicker (RBBF, *red*). Each vertical line represents a refresh of a frame, *tall lines* represent frames *with dots*. A *triangle* indicates that the frame also contains a triangle target in a to-be-attended *green area* or a false target in a to-be-ignored *red area*. The *short lines* indicate frames *without dots*. **c** A typical sequence of frames for the “ONE” (*green*) area to be attended (Color figure online)

The stimulus was generated by a Power MAC G4 using Matlab (Natick, MA) and the Psychophysics Toolbox (Brainard 1997; Pelli 1997), and displayed on a 19-inch monitor (Viewsonic PF790) with a vertical refresh of 120 Hz. It consisted of an annulus of fixed eccentricity (inner radius 5.45°; thickness 2.9°) divided into either two, four or eight regions of equal area, with half the regions colored green and the other half, red. The red and green luminance was adjusted psychophysically by brightness matching in a pilot study, to appear isoluminant with respect to each other. The red/green assignment was counterbalanced across trials and served to index the attended and ignored locations. The red and green regions were superimposed with an independent rapid visual serial presentation (RSVP) of visual search arrays consisting of 16 randomly positioned discs (distractors) 0.75° in diameter. The search array superimposed on the red regions was updated according to a random broadband flicker (rbbf). The search array presented on the green regions was updated regularly according to square wave flicker at one of three frequencies: 3, 8.6, and 20 Hz. An occasional search array contained a triangle target of the same area as a disc. Figure 1a shows three example frames depicting the three stimulus configurations. Figure 1b, c illustrate the flicker generation in a 50 s trial.

### Behavioral Task

Each participant went through a training session before the actual experiment. The attention instruction appeared at the beginning of each trial on the screen. The participants were asked to either “attend to green” or “attend to red”. This instruction required the participants to attend to the white disks in the colored region/s that they were asked to attend to and ignore the stimuli in the other colored region/s. Depending on the stimulus configuration, they were required to attend to one, two or four regions and respond by a button press, each time they spotted a white triangle in the attended region/s. They were asked to respond as quickly as possible. Only responses occurring between 150 and 1,000 ms were counted as valid.

There were, on average, 14 targets in a 50 s trial. In order to increase the spatial modulation of attention, in each trial there were three times as many frames containing triangles presented in the regions to be ignored as in those to be attended (Gobell et al. 2004). The participants were asked to “click when they were ready”, so that they had enough time to fixate at the center of the display and maintain fixation before the task began. The experiment consisted of 36 trials (3 stimulus configurations × 3 temporal frequencies × 2 attention instructions × 2 red/green phases that counterbalanced the red/green assignment). Thus there were two trials, counterbalancing color assignment, for each condition.

### EEG Recordings

Each subject was seated in front of the monitor displaying the stimulus, in a dark room. The electrophysiological data was obtained using a 128 channel geodesic sensor net, with the EEG electrodes placed on the observer’s scalp during the course of the experiment. Eight of those electrodes were disabled and the amplifier channels were used instead to record stimulus information using photocells directly attached to the monitor. Sixteen additional electrodes were removed due to the presence of artifact in some subjects, leaving 108 electrodes for the analysis. The EEG recording was sampled at a rate of 1,000 Hz, analog low-passed filtered at 50 Hz and mathematically referenced to the average of the 108 channels.

### Data Analysis

#### Behavioral Data

Behavioral responses that occurred within 150 and 1,000 ms after the onset of the target were counted as hits. Hits were calculated for each trial separately, generating a hit rate, calculated as the ratio of the number of hits to the total number of targets presented in that trial. Calculating dprime was complicated by the use of three times as many false targets as targets, but the essential effects reported here for hit rate were also observed for dprime. Hit rate was used as the performance measure in subsequent analysis of the behavioral and EEG data. A mixed-effects multi-way ANOVA was carried out for each attention condition, with temporal frequency, number of locations and gamer type as the independent variables (fixed factors) and individual subject as a random factor, and the hit rate as the dependent variable.

#### EEG Data

For each ~50 s trial, the EEG data were cropped so that, at each stimulus frequency, the input flicker data contained an integer number of cycles with a small prime number factor. This was done to increase the computational speed of the subsequent Fourier analysis on the EEG data. The integer number of cycles ensured that there was no spectral leakage. The stimulus frequency was centered on a bin of width approximately equal to 0.02 Hz. At each EEG channel, at every stimulus frequency, the SSVEP power was calculated as the squared amplitude of the Fourier coefficient of that frequency. The noise power was estimated as the 95th percentile value of the power in the surrounding 100 bins, 50 bins on either side of the flicker frequency (corresponding to ±1 Hz). SNR, calculated as the ratio of SSVEP power and the noise power, was the basic neural measure used to compare attention states across temporal frequencies and the number of attended/ignored locations. Two measures based on the SNR were obtained at each temporal frequency and stimulus configuration: SNR observed when the subject was attending to the flicker (SNRa) and the SNR observed when the subject was attending to the random broadband flicker (or ignoring the regular flicker, SNRu). The two red/green phases were averaged over, so that each stimulus configuration did not differ in the total area of visual stimulation.

#### Partial Least Squares Regression Analysis (PLS Analysis)

 The N-way toolbox (Andersson and Bro 2000) was used to fit a PLS model to the SNR data and the hit rate data. A separate model was generated for each gamer type, temporal frequency as well as attention condition. In the ‘Attend to Flicker’ condition, the model used the SNR responses to the attended flicker (SNRa) to fit the hit rate at the attended locations. In the ‘Attend to random broadband flicker’ condition, the model used the SNR responses to the ignored flicker (SNRu) to fit the hit rate at the attended locations. Each subject’s SNR value was normalized by the average across the different stimulus configurations at each EEG channel, in order to remove the variation in SNR due to the increasing number of attended/unattended locations. The mean SNR value across all subjects (within each gamer group) at each stimulus configuration was removed from the SNR value of each subject, separately for each channel. As a pre-processing step, the SNR values were subjected to a direct orthogonal signal correction or DOSC (Westerhuis et al. 2001) to remove the direction in the SNR that was orthogonal to the hit rate and that accounted for the largest variation in SNR. This led to a more efficient PLS model of the data using fewer components. The model in each condition was validated using a leave-one-out cross-validation routine. The fraction of variance in hit rate that was explained by the cross-validated model was the measure used to gauge the validity of the model. We used as many components as were required to explain at least 80 % of the hit rate data using the fitted model.

#### Statistical Analysis of SSVEP Dependence on Number of Attended/Ignored Regions

In order to determine if a significant monotonic trend could be observed in the EEG data, an ordered hypothesis test was used that converted the SNR value at every channel into a linear rank across the three stimulus configurations for each subject and temporal frequency. The ranks were then summed across all subjects, separately for each gamer category and temporal frequency. The sum, at every channel, was multiplied by a vector of ordered ranks (either [1 2 3] or [3 2 1]) to generate the non-parametric ordered-hypothesis test-statistic L (Page 1963) with 11° of freedom (12 subjects). This statistic was then compared with the critical values to determine whether or not the channel exhibited a monotonic (increasing or decreasing) trend as the number of regions increased. Only those channels were considered that showed an SNR above a certain threshold in either gamer group (SNR > 2 for 3 and 20 Hz; SNR > 4 for 8.6 Hz).

## Results

### Behavioral Results

The psychophysical measure used to evaluate the performance of the two groups of gamers was the hit rate, computed as the ratio of the number of correct responses to the triangle targets in the to-be-attended locations to the total number of triangles presented at those locations. The hit rate, both when the regular flicker was attended (e.g., ‘attend to green’) and when it was ignored, i.e., the random broadband flicker was attended (e.g., ‘attend to red’), was calculated for each trial and averaged across all twelve subjects in each experimental group.

**Fig. 2:**
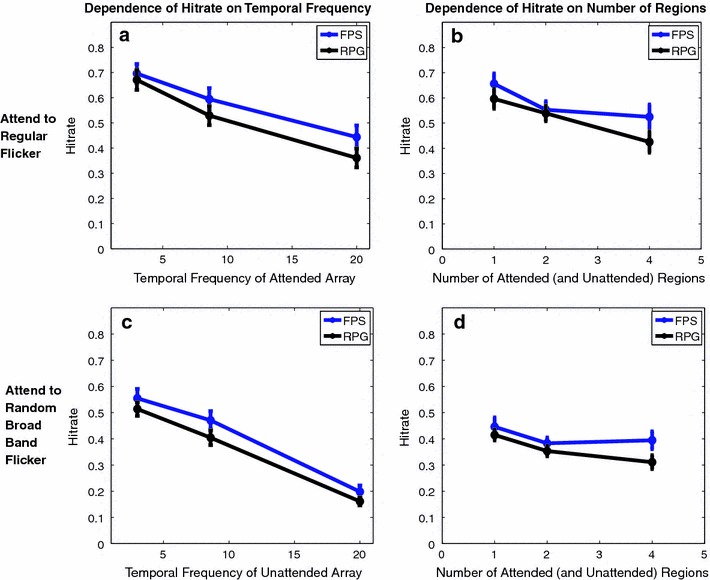
Behavioral Results: Hit rate, the fraction of detected targets, as a function of temporal frequency and of the number of attended regions for FPS and RPG players. **a** Hit rate at the attended locations (averaged over the number of locations) as a function of the temporal frequency of the attended locations. The unattended regions have Random Broadband Flicker, RBBF. **b** Hit rate (averaged over temporal frequencies) as a function of the number of regions to be attended. The number of attended locations always equals the number of unattended locations. **c** Hit rate (averaged over the number of attended locations) for RBBF as a function of the temporal frequency of unattended regions. **d** Hit rate for RBBF as a function of the number of attended regions (averaged over temporal frequencies of unattended regions). Both FPS players and RPG players exhibit similar trends, although the FPS players, on average performed the same or better than the RPG players

We found that when the regular flicker was attended, the hit rate in both groups of gamers decreased as a function of temporal flicker frequency of the stimulus (Fig. 2a). This was expected since the search array was updated at the stimulus flicker rate and therefore, the number of new frames/s to be processed also increased with temporal frequency (Ding et al. 2006). Since the attended and unattended locations were placed on the same peripheral annulus, the attention filtering was expected to be partial, resulting in an effect of the temporal frequency of the unattended search array on the hit rate at the attended locations. As illustrated in Fig. 2b, the hit rate did decrease as a function of the temporal flicker frequency at the unattended locations. A multi-way ANOVA, with temporal frequency (3, 8.6 and 20 Hz), number of attended/unattended locations (1, 2 and 4) and gamer type (FPS and RPG) as fixed factors and individual subject as a factor with random effects, was carried out separately for the two attention conditions (flicker attended and rbbf attended). As evident in Fig. 2, there was a main effect of temporal frequency when the regular flicker was attended (*F*_2,44_ = 86.56, *p* < 0.0001) and when the random broadband flicker was attended (*F*_2,44_ = 154.85, *p* < 0.0001).

Figure 2b illustrates the dependence of the hit rate on the number of attended/unattended locations when the regular flicker was attended (*F*_2,44_ = 30.16, *p* < 0.0001). Figure 2d illustrates the dependence of hit rate when the random broadband flicker was attended (*F*_2,44_ = 9.84, *p* < 0.0001). In both cases, there was an effect of the number of locations on the performance, with the RPG gamers showing a monotonic decrease in hit rate with the number of locations and the FPS gamers performing similarly when two or four regions had to be monitored.

As expected, the FPS gamers, on an average, always performed better or the same as RPG gamers (Green and Bavelier 2007). This effect of gamer type was not significant when the regular flicker was attended (*F*_1,22_ = 1.11, *p* > 0.05) nor when the random broadband flicker was attended (*F*_1,22_ = 1.97, *p* > 0.05). However, from Fig. 2b, d, it is evident that the biggest difference in performance between the two groups of gamers occurred when four regions had to be simultaneously attended (and ignored). A t test between the hit rates for the two groups of gamers at 4 regions demonstrated significantly higher hit rate for FPS players compared to RPG players (*p* < 0.05 for both ‘Attend to Flicker’ and ‘Attend to RBBF’). This result suggests that the difference in performance, as measured by hit rate, increases with task difficulty. Here, the increase in task difficulty comes both from increased crowding of attended and unattended region and from increased attentional demands, as the number of regions increased. Fast action gamers have been previously reported to have both an increased attentional capacity (Green and Bavelier 2003) as well as higher spatial resolution of vision (Green and Bavelier 2007), which presumably drives the better performance of the FPS players when four regions had to be attended.

### EEG Results

Signal to noise ratio of the SSVEP response to both attended and unattended regions of the display was the neurophysiological measure used to compare the neural strategies of the two groups of gamers. Specifically, we used the SNRa (SNR at the stimulus frequency when the flickering stimulus was attended) and the SNRu (SNR at the stimulus frequency when the stimulus was ignored while the broadband flicker was attended).

### Dependence of Spatial Distribution of SNR on the Temporal Frequency of the Stimulus

**Fig. 3:**
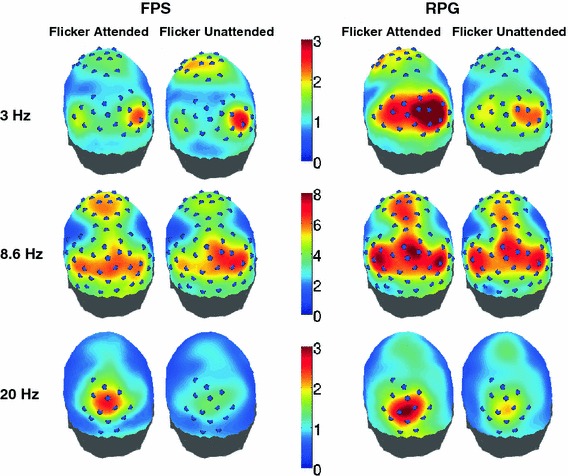
EEG results for FPS and RPG players averaged over the three stimulus configurations. The color (from *blue* to *red*) indicates the signal-to-noise ratio (SNR) in response to the regular flicker, as illustrated in the *central bars*. The occipital poles are on the bottoms of the flattened representations of the skull. The *blue discs* indicate channels with SNR subject to the following constraints: 3, 20 Hz, SNR > 2; 8.6 Hz, SNR > 4 (Color figure online)

The topographic plots in Fig. 3 illustrate the dependence of the SNR on the stimulus flicker frequency. As in previous studies (Srinivasan 1999; Srinivasan et al. 2006; Ding et al. 2006), the cortical areas that were entrained by a stimulus depended on the flicker frequency of the stimulus. For both categories of gamers, the 8.6 Hz flickering stimulus elicited the largest and most global responses, covering occipital, parietal and frontal cortex (Fig. 3, 8.6 Hz). The 3 Hz flicker also evoked large responses over parietal, occipital, and frontal cortex (Fig. 3, 3 Hz). The spatial distribution of the 3 Hz frontal cortex responses was similar to 8.6 Hz. The responses at 3 Hz over occipital and parietal cortex were distinct from the 8.6 Hz responses with foci away from the midline and somewhat right lateralized (not significant). In FPS players, these right occipital and parietal responses (averaged over stimulus configurations) were larger when the flicker was unattended. This was, however, not significant across subjects. Compared to the 8.6 and 3 Hz flicker, the 20 Hz flicker entrained a more local network in the visual cortex with responses mostly over occipital and parietal regions of the brain (Fig. 3, 20 Hz). The occipital/parietal response was focused on the midline, without the lateral responses seen at 8.6 and 3 Hz. The RPG players generally exhibited larger SNR values at each flicker frequency.

### Prediction of Hit Rate from Signal to Noise Ratio (SNR)

In order to examine the difference in neural strategy between the FPS and RPG players, we modeled the relationship between the SSVEP data at each temporal frequency (3, 8.6, and 20 Hz) and performance (hit rate) using a PLS (Bro 1996). We found predictive relationships only at 3 and 8.6 Hz but not at 20 Hz.

**Fig. 4:**
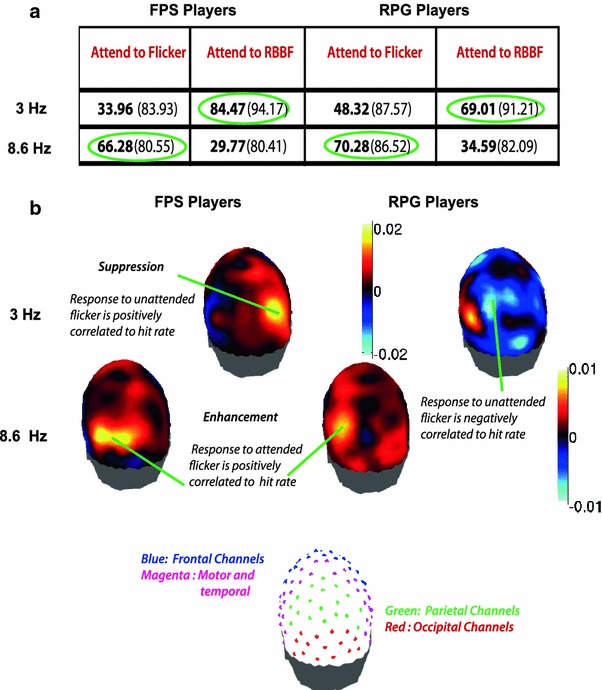
Summary of partial least squares (PLS) results in predicting psychophysical hit rates from SSVEP SNRs. **a** The percentage of between subject variance in hit rate accounted for by the PLS regression model. *Bold values* indicate the values associated with the cross-validated model; the values in *brackets* indicate the values associated with the fitted model. **b** Topographical representation of the regression coefficients of the PLS model. The colors represent the regression coefficients and indicate the extent and direction of correlation between the SNR and hit rate. At 3 Hz, the responses to the unattended stimuli predict hit rate, showing a positive correlation with hit rate in FPS players and a negative correlation in RPG players. At 8.6 Hz, the responses to the attended stimuli predict hit rate, showing a positive correlation with hit rate in both groups of gamers (Color figure online)

The independent variable was the SNR (at each electrode), in response to the regular flicker: (1) when it was attended and (2) when it was ignored. In each case, the corresponding dependent variable was the hit rate at the attended location/s which was updated according to: (1) a regular square wave flicker (regular flicker attended) and (2) random broadband flicker (regular flicker ignored). Figure 4a shows the percentage of variance in hit rate accounted for by the SNR as per the PLS model. The percentage of variance explained in hit rate is equivalent to an* r* ^2^ value. Figure 4b. shows the regression coefficients associated with each EEG channel. Positive value of regression coefficients indicated positive correlation between SNR and hit rate, while a negative value of the regression coefficients indicated a negative correlation between SNR and hit rate. The relative strength of the regression coefficients at each channel indicated the relative weighting of each EEG channel in the prediction of hit rate, as well as the extent of correlation with hit rate. Model validation was carried out using a leave one out cross-validation.

### 3 Hz Response to the Unattended Flicker Predicts the Hit Rate at the Attended Location

We found that the hit rate variation in response to the attended location/s was predicted by the SNR variation of the 3 Hz SSVEP to the unattended location/s (Fig. 4a, cross-validated values) in both groups of gamers. However the direction of correlation between the SNR and the hit rate, as reflected by the regression coefficients, distinguished the two groups. In the RPG players, the SNR at the unattended location/s was negatively correlated with the hit rate (Fig. 4b, 3 Hz). This is expected if signal enhancement is the mechanism being employed, since attention to the unattended location presumably takes away from the attention to the attended location. This model explained 69.01 % of the variance in hit rate. However, if the role of a cortical network were in the monitoring of the unattended locations, possibly to actively suppress the information at those locations, we would expect an increase in the SNR to be positively correlated with hit rate. This is what we observed in the FPS players (Fig. 4b, 3 Hz) with 84.47 % of the variance being explained by the model (Fig. 4a). In both groups of gamers, the responses over the parietal and occipital channels were most predictive of the hit rate, with the right parietal electrodes contributing the most to predicting hit rate in the FPS gamers.

### 8.6 Hz Response to the Attended Flicker Predicts the Hit Rate at the Attended Location

In the 8.6 Hz case, the variance explained in the hit rate by the leave one out cross-validated model indicated that the SNR of the SSVEP to the flicker at the attended location/s is predictive of the hit rate (Fig. 4a, cross-validated values). In both groups of gamers, SNR was positively correlated with hit rate (Fig. 4b, 8.6 Hz), with the SNR over left parietal electrodes showing the highest correlation. In the RPG players this model explained 70.28 % of the variance in hit rate, while in the FPS players the model explained 66.28 % of the variance. This positive correlation between the responses to the attended stimuli and the hit rate implies that information is selected from the to-be-attended locations by enhancing the inputs from these locations in both groups of gamers.

### Dependence of SNR on the Number of Attended/Unattended Locations

**Fig. 5:**
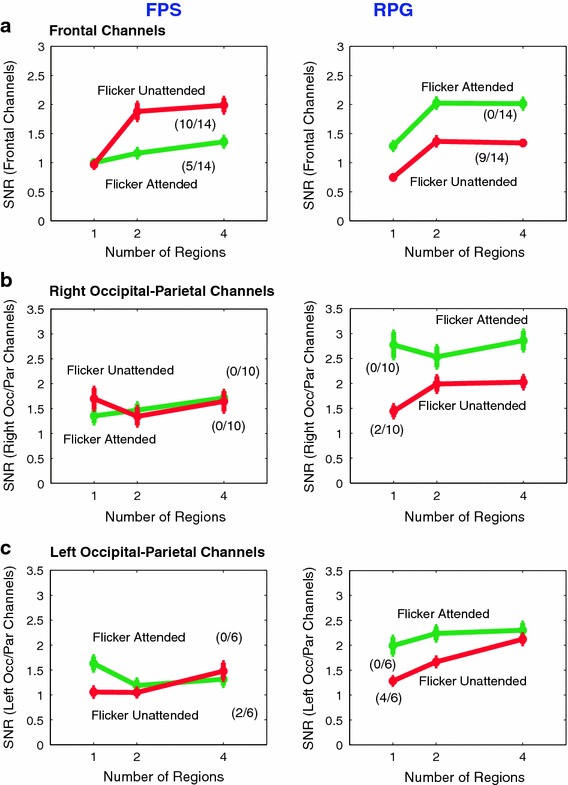
EEG results (3 Hz): The dependence of SNRa (flicker attended) and SNRu (flicker unattended) on the number of regions to be attended or ignored. SNRa and SNRu were averaged separately across the frontal (**a**), right parietal-occipital (**b**) and left parietal-occipital electrodes (**c**) shown in *blue* in Fig. 3 (1st row, 3 Hz). **a** In FPS players, the frontal SNRu > SNRa when two and four regions are simultaneously attended suggesting the role of active suppression in the selection of information. In RPG players, SNRa > SNRu for one, two and four regions. **b** The right occipital-parietal electrodes, in FPS players, show no significant monotonic trend. In RPG players, SNRu increases when more than one region has to be attended. **c** In FPS players, SNRu at the left occipital-parietal electrodes increases when four regions are attended, relative to when two regions are attended, with SNRa > SNRu only when one region is attended. In RPG players SNRu monotonically increases with number of regions to be attended or ignored (Color figure online)

**Fig. 6:**
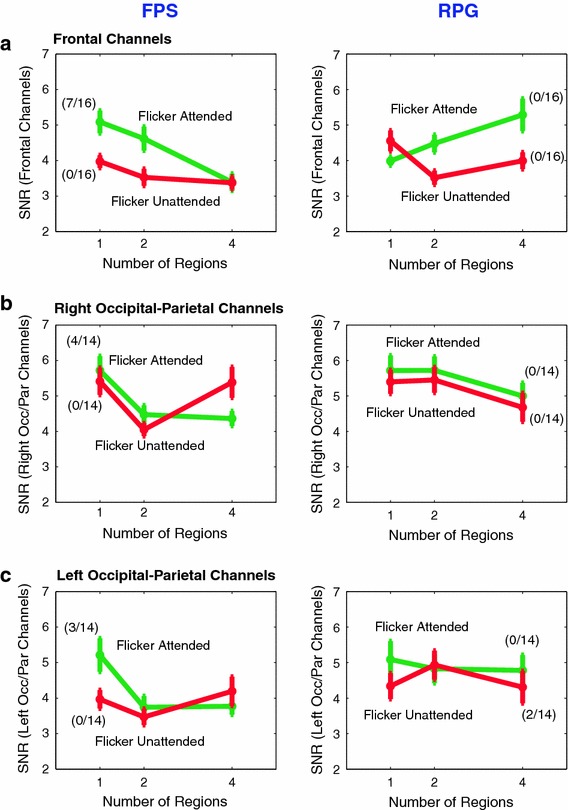
EEG results (8.6 Hz): The dependence of SNRa (flicker attended) and SNRu (flicker unattended) on the number of regions to be attended or ignored. SNRa and SNRu were averaged separately across the frontal (**a**), right parietal-occipital (**b**) and left parietal-occipital electrodes (**c**) shown in blue in Fig. 3 (2nd row, 8.6 Hz). **a** Over the frontal electrodes, in FPS players, SNRa decreases with increasing number of regions while in RPG players SNRa increases with increasing number of regions. **b** In FPS players, the SNRa over right occipital-parietal electrodes decreases with number of regions. In RPG players, these SNRa exhibits a small, non significant decrease when four regions are attended. **c** Similar to the electrodes on the right, the SNRa across the left occiptal-parietal electrodes also decreases with number of regions, in FPS players. In RPG players, the SNRa over these electrodes does not exhibit modulation with increasing number of regions, though the SNRu decreases when four regions are simultaneously attended (Color figure online)

**Fig. 7:**
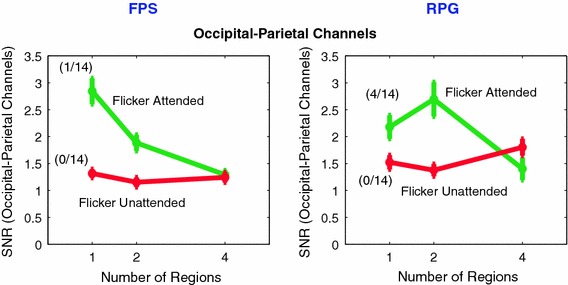
EEG results (20 Hz): The dependence of SNRa (flicker attended) and SNRu (flicker unattended) on the number of regions to be attended or ignored. SNRa and SNRu were averaged across occipital-parietal electrodes, shown in *blue* in Fig. 3 (3rd row, 20 Hz). Both groups of gamers exhibit similar trends at 20 Hz, SNRa decreases with increasing number of regions while SNRu does not show any significant monotonic trend (Color figure online)

We wanted to find how the SSVEP responses of the two groups of gamers differed as a function of the number of regions to be attended. Therefore, for each temporal frequency, we identified the electrodes that showed SNR above a certain threshold (SNR > 2 for 3 and 20 Hz; SNR > 4 for 8.6 Hz) in either gamer group for at least one of the attention conditions. These electrodes are displayed in Fig. 3 as blue circles on the topographical maps. The trends exhibited by subsets of these electrodes (frontal, occipital/parietal), as a function of number of regions, are displayed in Figs. 5, 6 and 7. On each plot, the fraction of electrodes, in each group, that showed significant monotonic trend in the illustrated direction, is displayed in brackets.

### 3 Hz Frontal Responses are Larger When Ignored Only in First Person Shooter (FPS) Players

In the 3 Hz flicker conditions, SNR at the frontal electrodes generally increased with increasing number of locations to be monitored, for both attention conditions and both gamer groups (Fig. 5a, 3 Hz). In both gamer groups, SNRu (response to the unattended flicker) increased when the number of regions to be attended (and ignored) increased from 1 to 2 or 4 locations. In the RPG players, the SNRa (response to the attended flicker) also followed a similar trend, while in the FPS players the SNRa continued to increase even when 4 locations had to be monitored. The fraction of channels that exhibited this significant monotonic trend is displayed in brackets next to the trend lines. The main differences between the two groups of gamers were in the relative strengths of the SNRa and SNRu. In RPG players, the SNRa was always larger than the SNRu. In the FPS players, the SNRu was larger than the SNRa when more than one location had to be attended/ignored. The higher SNRu (averaged over significant monotonic electrodes in FPS players), relative to SNRa approached but did not reach significance (*p* = 0.0576). This increased response to the unattended locations exhibited by the FPS players again points to an active mechanism of suppression of unattended information, possibly originating in the frontal cortex. That an increased response to the unattended locations was observed only when more than one location had to be attended and ignored further supports our claim of active suppression because subjects are more likely to benefit from suppression in conditions where attended and ignored regions are interleaved.

In RPG players, a subset of left and right occipital-parietal electrodes also showed significant increasing SNRu with number of regions (Fig. 5b, c). In FPS players, on the other hand, the right occipital-parietal electrodes did not show a monotonic trend in SNRu (Fig. 5b), though a small subset of left occipital-parietal electrodes showed a significant monotonic increase in SNRu (Fig. 5c). Also noteworthy, is the fact that in the RPG players, the responses to the attended flicker were always higher than the responses to the unattended flicker even over left and right occipital-parietal cortices. The FPS players, on the other hand, showed lesser/no differences between the responses to the attended and unattended flickers over occipital-parietal cortices compared to those over the frontal cortex.

### Direction of the Variation in the 8.6 Hz Frontal Responses to the Attended Flickering Stimulus Depends on the Gamer Type

The modulation of the 8.6 Hz frontal responses with the number of regions to be attended and ignored, distinguished the two groups of gamers. When the flicker was attended, the SSVEP amplitude produced by the flickering stimulus decreased as a function of the number of locations in the FPS gamers, whereas in the RPG gamers these responses increased (Fig. 6a, 8.6 Hz). As described above, because an increase in the strength of the SSVEP responses with task difficulty suggests a compensatory mechanism, it is possible that in RPG players, the 8.6 Hz responses were responsible for enhancing the information at the attended locations. In FPS players, the decreased responses indicate the effect of the increased attentional demands on the neural resources, with no evidence of compensatory gain. The right and left occipital-parietal electrodes in FPS gamers also showed significant decreasing trend in SNRa similar to the frontal electrodes (Fig. 6b, c). On the other hand these electrodes in RPG gamers showed a non significant but opposite trend to the frontal electrodes (Fig. 6b, c).

### The Variation of Local 20 Hz Responses with Number of Locations is Similar in both Gamer Groups

In both groups of gamers, SSVEP responses to the attended flickering stimulus decreased with increasing number of regions to be attended and ignored (Fig. 7, 20 Hz). These local responses, over occipital and parietal cortices, reflect the low pass characteristics of attention (Gobell et al. 2004). In the RPG gamers, SNR increased slightly when two regions were attended relative to when only one region was attended and then decreased when four regions were simultaneously attended. In both groups of gamers, there is no difference between the responses to the attended or ignored flickering stimulus when 4 regions had to simultaneously monitored, indicating that there was no effect of attending to the flicker in that condition. This lack of attention modulation is consistent with the poor performance (low hit rates) in this condition (see Fig. 2a, c).

The monotonically modulated frontal responses in the 3 and 8.6 Hz cases are indicative of the compensatory mechanisms that follow the increasing task demands. The occipital/parietal responses that track hit rate (as observed with the PLS modeling) presumably indicate the success of the mechanisms coupled to the processes in the frontal cortex.

## Discussion

Two neural strategies that can be employed to select information from a subset of locations in space are the enhancement of information at the attended location (Hillyard et al. 1998; Carrasco et al. 2000) and the suppression of information at the unattended locations (Dosher and Lu 2000; Serences et al. 2004). The FPS players and the RPG players differed in the neural strategies employed to selectively attend to multiple non-contiguous regions in space. We found that RPG players use enhancement of the attended information in order to select the information at the attended location. The FPS players, on the other hand, seem to be using both enhancement as well as the suppression of the information at the unattended location to mediate selective attention.

### A 3 Hz network mediates selective attention in the FPS players by suppressing unattended information

Using the partial least squares model to predict hit rate from SNR, we found that the 3 Hz SSVEP response to the search array at the unattended locations was the best predictor of hit rate. This was true in both groups of gamers. In RPG players, the SNR responses were *negatively* correlated with hit rate, as would be expected of a network involved in signal enhancement at the attended locations. Any increases in the responses of this network to the unattended locations would be indicative of attention wandering to the other flicker that would be reflected in lower hit rates. However, in FPS players, the responses to the unattended stimuli were *positively* correlated with the hit rate at the attended location. This suggests the possible role of this network in actively suppressing the information at the unattended locations resulting in the increased response to the unattended stimuli being accompanied by higher hit rates. Moreover, in the FPS players, the electrodes showing the highest correlations were over the right parietal and temporal cortices. These regions have been previously implicated in the monitoring of the unattended locations for behaviorally relevant information (Corbetta et al. 2008; Serences and Yantis 2006).

In addition to a parietal-temporal network in which responses to unattended stimuli were positively correlated with hit rate at the attended location, the presence of an active ‘suppression network’ was also indicated by the monotonically modulated frontal SSVEP responses to the attended and unattended stimuli. The FPS players exhibited an increased frontal response to the unattended locations relative to the attended locations, only when two and four regions had to be simultaneously attended. This interaction suggests an increased processing of the unattended stimuli by the frontal cortex, when more than one location had to be ignored. The PLS results confirm that this increase possibly reflects a mechanism of active suppression. The RPG players, on the other hand, did not exhibit greater responses to the unattended locations irrespective of the number of locations to be monitored, although, like the FPS players, their responses to the attended and unattended stimuli increased with increasing number of locations. This increase in responses to the unattended location/s in the RPG players could also reflect a compensatory active suppression mechanism. However, even if the RPG players were attempting to suppress the unattended locations, unlike in the case of FPS players, there is no robust evidence that were successful in using that strategy to perform the behavioral task, since their parietal responses to the unattended 3 Hz flicker was negatively correlated with hit rate.

### A 8.6 Hz Network Mediates Selective Attention, by Signal Enhancement at the Attended Locations

The results of the PLS analysis demonstrate that in the 8.6 Hz case, the responses to the stimulus at the attended location was the best predictor of the hit rate at the attended location/s. Since these responses were positively correlated with hit rate, it is likely that the network of cortical areas tagged by the 8.6 Hz stimuli were responsible for the enhanced representation of the attended stimuli (or signal enhancement at the attended locations). In both groups of gamers, the cortical areas exhibiting the highest correlation were regions over the parietal and occipital cortices, especially in the left hemisphere. Thus, at 8.6 Hz, there was evidence of signal enhancement in both groups of gamers. In the RPG players, this was further confirmed by the trend of the attended frontal responses with increasing number of locations. The attended frontal responses, *increased* with increasing number of locations, suggesting a compensatory mechanism for the increase in attentional demands and task difficulty. These PLS results suggest that this frontal compensatory mechanism enhances the signal at the attended location by increasing its representation.

In the FPS players, on the other hand, the 8.6 HZ attended frontal responses *decreased* as a function of the increasing number of locations. In the case of these gamers, the 8.6 Hz frontal responses seem to have less of a role in compensating for the increased attentional load. Rather, these frontal responses reflect the decreased attentional modulation that occurs with increasing the number of attended and ignored regions (Gobell et al. 2004). However, the PLS results still demonstrate a positive correlation between occipital and parietal responses to the attended stimuli and the hit rate at those locations, arguing for the FPS gamers also using signal enhancement as a possible additional mechanism for selecting information.

### A 20 Hz Network Reflects the Low Pass Spatial Filter Characteristics in Both Gamer Groups

As the number of locations to be attended/ignored increased, the spatial frequency of the required attention distribution also increased. This resulted in a decrease in the relative enhancement of the information in the attended areas and the relative suppression of that in the unattended areas (Gobell et al. 2004). According to Gobell et al. (2004), such a decrease in the modulation of attention can be modeled as a low pass spatial filter. The decrease in attention modulation was evident in the decrease in the rate of target detection. In the present study also, the hit rate decreased when more than one location had to be simultaneously attended to. This decreased attention modulation was more or less mirrored in the 20 Hz responses to the attended flicker for both groups of gamers. The 20 Hz network is a relatively local network most likely to have properties reflecting properties of visual cortex, which receives both direct visual input as well as feedback from the parietal and frontal cortices. Therefore, the responses of this network as a function of task difficulty would reflect the composite attention effects, taking into account both the properties of the stimulus (which dictates the required attention distribution), as well the top down signals related to the attention instructions.

### Distinct Functional Networks are Evident at Different Frequencies

SSVEPs depend strongly on the physical properties of the stimulus and the brain networks that are synchronized by the input frequency. A brain network will “resonate” with and provide strong responses at frequencies that match the combined effects of intrinsic time constants (e.g., rise and decay time of post synaptic potentials) and the transmission delays between brain areas. Consistent with this view are the observations that (1) SSVEPs at low frequencies (<15 Hz) have a spatial distribution extending to temporal and frontal areas that strongly depend on flicker frequency, suggesting spatio-temporal resonance (Ding et al. 2006; Srinivasan et al. 2006), and (2) at higher flicker frequencies (>15 Hz) the responses are localized to occipital/parietal areas as the flicker is too rapid to synchronize distant areas with longer transmission delays. Thus, each of our flicker frequencies entrains a different network that is differentially modulated by attention. Additionally, these functionally distinct networks are likely operating in conjunction irrespective of whether or not they are ‘tagged’ or entrained by the stimulus flicker frequency in a specific trial. Ding et al. (2006) systematically studied SSVEPs at frequencies ranging from 3 to 20 Hz, and found frequencies where SSVEPs were enhanced by attention, frequencies where SSVEPS were enhanced when the stimulus is not attended (suggesting active mechanisms of suppression) and frequencies where no effect of attention on the SSVEP was observed. The results of the present study are consistent with the idea of different functional networks observed at each frequency.

### Performance Differences Between FPS and RPG Players

Using mixed effects ANOVA with subject as a random factor, we expected, but did not find a significant effect of gamer type on hit rate. However, a *t* test comparing hit rate between the two gamer groups in the four regions case showed a significantly higher hit rate for the FPS players compared to RPG players. This suggests that when the task was easier, when only one and two regions had to be simultaneously attended, the FPS players didn’t enjoy a significant advantage over RPG players. However, as the task difficulty as well as the stimulus complexity increased, when four regions had to be attended, they performed significantly better. Thus, it is perhaps reasonable to infer that though FPS players and RPG players seem to use different neural attentional strategies of selection, the neural strategy used by FPS players is most advantageous when the task is especially difficult.

## Summary and Conclusion

Our findings show that it is possible to infer the strategy that subjects use to perform a search task from the profile of their brains’ SSVEP response to the search stimuli. The SSVEP data suggest that visuo-spatial training such as that provided by playing FPS games, could improve performance on demanding visual search tasks by modifying the neural strategy of selective attention. FPS players, in addition to signal enhancement strategies, appeared to employ an active suppression mechanism that is not detectible in RPGs who appear to use only a signal enhancement mechanism to selectively attend to multiple interleaved regions in visual space. The SSVEP data suggest that fast-action video gaming trains the mechanisms of suppression of irrelevant information to improve performance in a rapidly changing complex environment.
